# Complete Genome Sequence of *Wohlfahrtiimonas chitiniclastica* Strain BM-Y, Isolated from the Pancreas of a Zebra in China

**DOI:** 10.1128/genomeA.00643-16

**Published:** 2016-06-30

**Authors:** Wei Zhou, Mu Li, Lingwei Zhu, Fuyou Hua, Xue Ji, Yang Sun, Jun Liu, Xuejun Guo

**Affiliations:** a Institute of Military Veterinary, AMMS, Key Laboratory of Jilin Province for Zoonosis Prevention and Control, Changchun, China; b Shenzhen Safari Park, Shenzhen, China

## Abstract

Here, a complete genome sequence of *Wohlfahrtiimonas chitiniclastica* strain BM-Y is presented. The whole genome is 2.18-Mb and contains a *bla*_VEB-1_ gene cassette which endows it with resistance to ceftazidime, ampicillin, tetracycline, etc. To our knowledge, this is the first time that an extended spectrum beta-lactamase (ESBL) type *W. chitiniclastica* strain has been found.

## GENOME ANNOUNCEMENT

*Wohlfahrtiimonas chitiniclastica*, the type species of the *Wohlfahrtiimonas* genus, is a Gram-negative bacillus with close phylogeny to *Ignatzschineria larvae*, and is also a pathogen of myiasis in homeless adults, *Odocoileus virginianus*, and cattle (1–4). Although the pathogenesis of *W. chitiniclastica* associated disease has not been interpreted completely, the fly *Wohlfahrtia magnifica* acting as a carrier of *W. chitiniclastica* and wound contamination was believed to be the key factor of bacterial septicemia (5). To date, five *Wohlfahrtiimonas* species and no more than 20 *W. chitiniclastica* strains have been isolated in the world. Of these, only antibiotic sensitive strain SH04 has been sequenced completely in China (6).

Given that *W. chitiniclastica* can cause severe infections in clinics and threaten the health of humans and animals, here, we report and analyze the complete genome sequence of *W. chitiniclastica* strain BM-Y, which was isolated from the pancreas of a zebra in China. BM-Y carried a *bla*_VEB-1_ gene cassette resistant to ceftazidime, ampicillin, tetracycline, etc. These characterizations were found in *W. chitiniclastica* first.

Whole-genome sequencing of *W. chitiniclastica* strain BM-Y was performed using a PacBio RS II system with a 20-kb library and C4-P6 chemistry. A total of 187,537 long circular reads (314-fold coverage) with an average length of 3,670 bp and 82.6% estimated accuracy were used as input for genome *de novo* assembly by single-molecule real-time (SMRT) analysis pipeline v2.3.0 in conjunction with RS_HGAP_Assembly3 protocol (7). Prediction and annotation of coding sequences were conducted with Glimmer software, Swiss-Prot, KEGG, COG, and GenBank nr databases. Transfer RNAs (tRNAs) were predicted using tRNAscan-SE (8) and ribosomal RNAs (rRNAs) were predicted using RNAmmer (9). Gene content was analyzed using BLASTp with parameters of more than 50% sequence identity and an e-value cutoff of less than 1e−5. Whole chromosome alignment and comparison was conducted with MUMmer3 (10).

The genome of *W. chitiniclastica* strain BM-Y consisted of a chromosome of 2,181,980 bp (two contigs) with 43.50% G+C content. Preliminary draft annotation indicates that the genome is predicted to contain 2,085 genes encoding for 1,856 proteins and 55 for tRNA. Reciprocal average nucleotide identity (ANI index) between BM-Y and two reference strains, SH04 and DSM18708, reveals that BM-Y shares 98.20% and 97.05% identity with SH04 and DSM18708, respectively, while SH04 and DSM18708 share 97.21% identity. Furthermore, BM-Y chromosome alignments at the nucleotide level with genomes of strains SH04 and DSM18708 reveal large chromosome inversions and rearrangements; the *bla*_VEB-1_ gene cassette is contained within contig2.

### Nucleotide sequence accession numbers.

The *W. chitiniclastica* strain BM-Y whole-genome shotgun project has been deposited at DDBJ/ENA/GenBank under the accession number LVXD00000000. The version described in this paper is version LVXD01000000.
